# Transcription Factor NF-κB Is Transported to the Nucleus via Cytoplasmic Dynein/Dynactin Motor Complex in Hippocampal Neurons

**DOI:** 10.1371/journal.pone.0000589

**Published:** 2007-07-11

**Authors:** Ilja Mikenberg, Darius Widera, Aljoscha Kaus, Barbara Kaltschmidt, Christian Kaltschmidt

**Affiliations:** Institut für Neurobiochemie, Universität Witten/Herdecke, Witten, Germany; Laboratory of Neurogenetics, National Institutes of Health, United States of America

## Abstract

**Background:**

Long-term changes in synaptic plasticity require gene transcription, indicating that signals generated at the synapse must be transported to the nucleus. Synaptic activation of hippocampal neurons is known to trigger retrograde transport of transcription factor NF-κB. Transcription factors of the NF-κB family are widely expressed in the nervous system and regulate expression of several genes involved in neuroplasticity, cell survival, learning and memory.

**Principal Findings:**

In this study, we examine the role of the dynein/dynactin motor complex in the cellular mechanism targeting and transporting activated NF-κB to the nucleus in response to synaptic stimulation. We demonstrate that overexpression of dynamitin, which is known to dissociate dynein from microtubules, and treatment with microtubule-disrupting drugs inhibits nuclear accumulation of NF-κB p65 and reduces NF-κB-dependent transcription activity. In this line, we show that p65 is associated with components of the dynein/dynactin complex in vivo and in vitro and that the nuclear localization sequence (NLS) within NF-κB p65 is essential for this binding.

**Conclusion:**

This study shows the molecular mechanism for the retrograde transport of activated NF-κB from distant synaptic sites towards the nucleus.

## Introduction

Rel/NF-κB proteins are a family of transcription factors involved in regulating the expression of genes relevant in a wide range of different cellular processes, such as apoptosis and cell survival, stress and immune response, differentiation and proliferation [1], [2]. NF-κB acts as a dimer consisting of various combinations of five subunits including p65 (RelA), c-Rel and RelB, containing transcriptional activation domains, and p50 and p52, lacking transcriptional activation domains. The heterodimer p50/p65 is predominant in the most cell types [3], [4]. In an inactive state, NF-κB is retained in the cytoplasm by inhibitors of NF-κB (IκBs), of which IκBα and IκBβ are the most abundant. IκBs act by masking nuclear localization signals (NLS) within NF-κB subunits. A classical NLS consists of a stretch of basic amino acids, arginines and lysines [5]. Classical NLS is found in p50 and p65 [6]. Upon activation, IκB is phosphorylated by an IκB kinase complex, which leads to ubiquitination and proteosomal degradation of IκB. As a consequence, the NLSs of p50 and p65 are unmasked, and the dimers are translocated into the nucleus, where they initiate transcription by binding regulatory DNA sequences of responsive genes.

Initially NF-κB has been the object of intense studies in the immune system, where it is involved in regulation of the host defense and inflammation [4]. A growing amount of experimental data supports the present view that NF-κB is involved in neural-specific functions extending beyond immune and inflammatory responses, as synaptic plasticity, learning and memory [7]–[11]. Within the nervous system, NF-κB is widely expressed [12], [13] and is activated by a variety of neurotransmitters and neurotrophic factors [14]–[17]. NF-κB has been associated with synaptic plasticity since it is present in pre-and postsynaptic sites and can be locally activated in synapses [18], [19]. Interestingly, exclusively p65/p50 dimer was detected in isolated synaptosomal preparations [7], [18], [19]. Moreover, signals specific to the nervous system, such as glutamate receptor binding and membrane depolarization induce NF-κB activation in hippocampal and cerebellar granule neurons in cell culture [17], [20]. Blockade of NMDA receptors and L-type Ca^2+^ channels was shown to effectively inhibit basal synaptic activity of NF-κB [7], [15], [20]. These results suggest that neuronal NF-κB activity to be controlled by the level of intracellular Ca^2+^. Consistent with this data three cellular sensors of the cytosolic Ca^2+^ levels calmodulin, protein kinases C (PKCs), and the p21^ras^/phosphatidylinositol 3-kinase (PI3K)/Akt pathway are demonstrated to be simultaneously involved in the steps linking the Ca^2+^ second messenger to NF-κB activity [20]. Moreover, at least in mature hippocampal neurons, the Ca^2+^-dependent pathway triggering activation of NF-κB requires CaMKII [7]. Results obtained with learning experiments in animal models support the idea of NF-κB as a significant component of the molecular mechanism of memory formation. Pharmacological inhibition of NF-κB and administration of κB decoy DNA induced memory impairment in crab and mice [21], [22]. Mice lacking the p65 subunit of NF-κB showed impaired spatial learning [7], [9], [11]. A study on fear conditioning demonstrated requirement for activation and acetylation of NF-κB in rat amygdala for long-term memory consolidation [23], [24]. Pre-treatment of hippocampal slices with κB decoy DNA prevented induction of LTD and significantly reduced the magnitude of LTP [25].

In hippocampal neurons p65-GFP fusion protein is shown to redistribute from distal processes to the nucleus after glutamate or kainate stimulation in a retrograde way by Wellmann and coworkers [26]. In further studies with photobleaching Meffert and colleages show that NF-κB movement within dendrites is directed in the retrograde direction [7]. To our knowledge, the mechanism allowing activated NF-κB located in distal neuronal compartments to be transported to the nucleus have not been studied previously. The elongated morphology of neurons poses a particular challenge to intracellular signal transduction. Signals generated at the synapse must be transported over long distances to the nucleus, where they can induce changes in gene expression. The most efficient mechanism for intracellular long-distance transport involves the association of a transcription factor with molecular motors that move along the cytoskeleton. There are several studies demonstrating that NLS-containing proteins are delivered to the area of the nuclear membrane along microtubules via the molecular motor dynein [27], [28].

In this study, we provide evidence suggesting the requirement of dynein/dynactin motor complex in retrograde transport of NF-κB following synaptic activation of hippocampal neurons. We show that the p65 subunit of NF-κB is associated with dynein and with members of the dynactin family of motor-associated proteins. Disruption of microtubule function by depolymerisation with colchicine or vincristine impaired accumulation of NF-κB in the nucleus following glutamate stimulation. Moreover, both of these drug treatments reduced NF-κB-dependent transcription activity. Similar results were obtained by overexpression of dynamitin, which is known to inhibit cytoplasmic dynein function by disrupting dynactin, further supporting the idea that dynein functions in p65 transport. In addition, we show that p65 is associated with components of dynein/dynactin complex *in vivo* and *in vitro* and that NLS is essential for this binding.

## Results

### NF-κB and dynein/dynactin molecular motor exist in a complex *in vivo*

**Figure 1:**
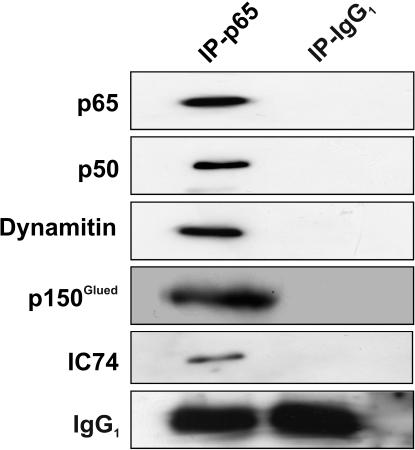
NF-κB exists in a complex with dynein/dynactin *in vivo.* NF-κB p65 co-immunoprecipitates with dynactin and cytoplasmic dynein. 10 mg soluble rat brain extract were cross-linked with DSP to stabilize transient protein-protein interactions and subsequent immunoprecipitated with an anti-p65 antibody. Immunoprecipitates were analyzed by western blotting with an anti- NF-κB p65 or NF-κB p50 antibody or with antibodies against dynein intermediate chain (IC 74) or the motor-associated proteins dynamitin and p150^Glued^. Control IP was done with a non-immune monoclonal antibody.

To investigatethe association of NF-κB and dynein/dynactin complex, we performed immunoprecipitation from rat brain extracts with an antibody directed against the p65 subunit of NF-κB (Figure 1). We next carried out a western blotting screen of the immunoprecipitates for proteins reported to be essential components of multisubunit dynein/dynactin motor complex. NF-κB p65 was found to coprecipitate with the intermediate chain of cytoplasmic dynein (IC74) and with two members of the dynactin family: dynactin p50 (Dynamitin) and p150^Glued^. The binding is specific since no bound proteins are detected with the control non-immune antibody. Dynactin, a multiprotein complex with distinct microtubule- and cargo-binding domains, is an integral part of the cytoplasmic dynein motor and is required for dynein-based motility [29], [30]. The largest subunit of dynactin, p150^Glued^, binds the dynein intermediate chain and has an N-terminal microtubule-binding domain. p150^Glued^ is expressed in multiple neurons and is a required activator of cytoplasmic dynein-mediated retrograde transport [31]–[34].

### The functional nuclear localization signal (NLS) of p65 is essential for its interaction with dynein/dynactin *in vitro*

**Figure 2:**
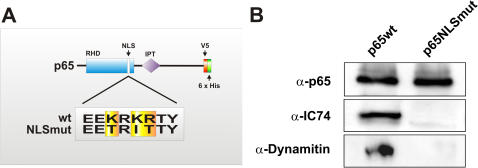
The functional nuclear localization signal (NLS) of p65 is essential for its interaction with dynein/dynactin *in vitro.* (A) Nuclear localization signal (NLS) of wild-type NF-κB p65 and its NLS mutant (NLSmut). The NLS of NF-κB p65 consists of a stretch of a basic amino acids, arginines and lysines. The three point mutations were introduced into the NLS (depicted in yellow). The construct was tagged with polyhistidine to enable purification from bacterial extracts and pull-down assays. (B) Polyhistidine-tagged wild-type p65 and p65 with mutant NLS (NLSmut) purified from bacterial extracts were immobilized separately on nickel-coated matrix and incubated with the rat brain extract. The bound material was eluted from the matrices, fractionated by SDS–PAGE and examined by Western blot with anti-IC74 and anti-dynactin p50 antibodies. As expected, wild-type p65 associates with dynein/dynactin *in vitro.* In contrast, p65 with mutant NLS failed to form this complex.

In eukaryotic cells, transport of proteins into the nucleus is mediated by short amino acid sequences that are reffered to as nuclear localization signals (NLS). Classical NLS-dependent nuclear transport is dependent on transport factors that belong to the importin families of proteins [35]. Importin α binds NLS directly, and its affinity for NLS is increased by interaction with importin β. Importins are found throughout axons and dendrites and play a critical role in transporting synaptically generated signals towards the nucleus [36]. The trimeric import complex containing the NLS-bearing cargo, importin α and importin β was found to traffic retrogradely due to an interaction of importin α with the motor protein dynein [37]. These findings are consistent with our previous studies demonstrating that in hippocampal neurons the retrograde transport of EGFP-p65 was dependent on functional NLS [26]. We created a p65 construct with a mutated NLS containing three point mutations to assess the ability of this mutant to associate with dynein/dynactin complex (Figure 2A). Polyhistidine-tagged wild-type p65 and p65 with mutant NLS (NLSmut) purified from bacterial extracts were immobilized separately on nickel-coated matrix and incubated with the rat brain extract. The bound material was eluted from the matrices, fractionated by SDS–PAGE and examined by Western blot with anti-IC74 and anti-dynactin p50 antibodies (Figure 2B). As expected, wild-type p65 associates with dynein/dynactin *in vitro.* In contrast, p65 with mutant NLS failed to form this complex. These results demonstrate a functional link between the presence of a nuclear localization signal and dynein-dependent transport.

### Inhibition of dynein function by overexpression of dynamitin impairs nuclear accumulation of NF-κB and reduces NF-κB-dependent transcription activity

**Figure 3:**
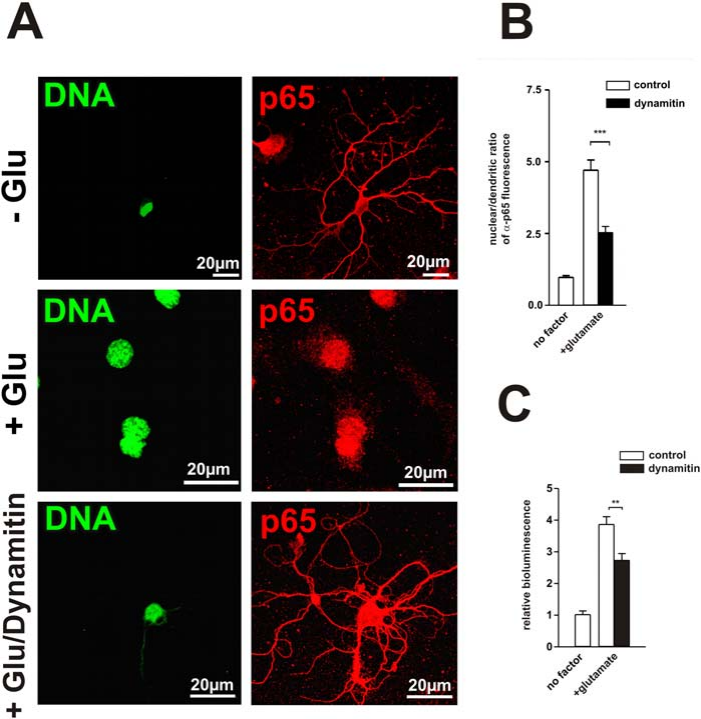
Inhibition of dynein function by overexpression of dynamitin impairs nuclear accumulation of NF-κB and reduces NF-κB-dependent transcription activity. (A) Cultured hippocampal neurons transfected with dynamitin or mock vector were left untreated or incubated with 300 µM glutamate for 5 min. After 90 min neurons were fixed and analyzed for subcellular NF-κB p65 destribution. Nuclei were stained with SYTOX (green). Anti-p65 immunoreactivity is depicted in red. (B) Quantification of nuclear/dendritic ratio of α-p65 fluorescence in dynamitin-transfected and control-transfected neurons with and without glutamate stimulation. Note that transfection with dynamitin impaired redistribution of NF-κB p65 from distal sites to the nucleus following glutamate stimulation. Fluorescence measurements were made from 20 to30 neurons in each experimental condition. (C) Reporter gene assay showed reduced NF-κB-dependent transcription activity in neurons overexpressing dynamitin.

If cytoplasmic dynein is required for the transport of NF-κB to the nucleus, we would expect inhibition of dynein function to impair nuclear accumulation of NF-κB and NF-κB-dependent transcription. To test this hypothesis, we overexpressed dynamitin (p50 dynactin) in cultured hippocampal neurons. Overexpression of dynamitin causes a dissociation of dynactin and inhibits multiple forms of dynein-mediated transport [38]–[39]. Thus, dynamitin overexpression is a powerful tool to examine the role of dynein and dynactin in the retrograde NF-κB transport. First, we analyzed the subcellular redistribution of NF-κB p65 as response to a glutamate stimulus. Unstimulated neurons were shown to have high levels of activated nuclear NF-κB due to basal synaptic transmission [40], which at least partially can be suppressed by pharmacological inhibitors of action potential generation, glutamate antagonists and L-type Ca^2+^ channel blockers [7]. For this reason we treated neurons with activity antagonists APV, CNQX and nimodipine 24 h prior to glutamate stimulation. Interestingly, cultures with inhibitor preincubation had potentiated NF-κB activation after glutamate stimulation. We interpreted this effect as increased synaptic strength in compensation for lost synaptic activity. In subsequent experiments, preincubation with inhibitors mentioned above was used to block basal NF-κB activity and to provide stable baseline and maximal response. Additionally, Leptomycin B, an inhibitor of nuclear export, was used during the stimulation to trap p65 that reached the nucleus, thus permitting small amounts to be more readily detected. To inhibit newly synthesized IκBα to transport NF-κB back to the cytoplasm, neurons were exposed to 30 µM anisomycin for 1h before and during experiments. Freshly isolated and dissociated hippocampal neurons were transfected with dynamitin or mock vector and after plating left to mature for 8 days in culture. This culturing time was shown to be sufficient for neurons to develop mature processes and high level of synaptic connectivity. At 8 days in culture, mature neurons were subjected to a 5 min pulse with 300 µM glutamate. Cells were then washed and placed back in an incubator. To determine the optimal assay run time to achieve maximal NF-κB nuclear accumulation, neurons were incubated up to 3h. The maximum response was achieved at 90 min. These observations are in general agreement with previous studies [26]. After this incubation time neurons were immunostained for p65 (Figure 3A). As expected, in unstimulated neuronal cultures p65 was mainly localized within the cytoplasm, including proximal and distal neuronal processes. In contrast, glutamate treatment resulted in redistribution of NF-κB from distal processes to the nucleus. Consistent with the hypothesis of dynein-dependent NF-κB transport, overexpression of dynamitin nearly completely blocked this relocation. Figure 3B shows that there was a significant reduction of nuclear/dendritic ratio of NF-κB p65 immunofluorescence intensity in dynamitin-transfected and glutamate-stimulated cultures compared with mock-transfected, glutamate-stimulated neurons. For functional analysis, we used a reporter gene assay to further evaluate the effect of dynein inhibition on NF-κB transport by dynamitin overexpression (Figure 3C). Firefly Luciferase has a half-life of only 3 h in mammalian cells, making it suitable to monitor expression dynamics over long period of time without limitation, owing to the accumulation of residual luciferase. Because of this, we could detect rapid increases or decreases in neuronal gene expression even weeks after reporter gene transfection. Luciferase assays were performed using the Dual Luciferase Assay System (Promega). Glutamate application increased NF-κB-dependent activity nearly 4-fold compared to untreated controls. Co-transfection of the dynamitin expression plasmid significantly decreased the activity measured.

### Microtubule-perturbing drugs inhibit neuronal transport of NF-κB

**Figure 4:**
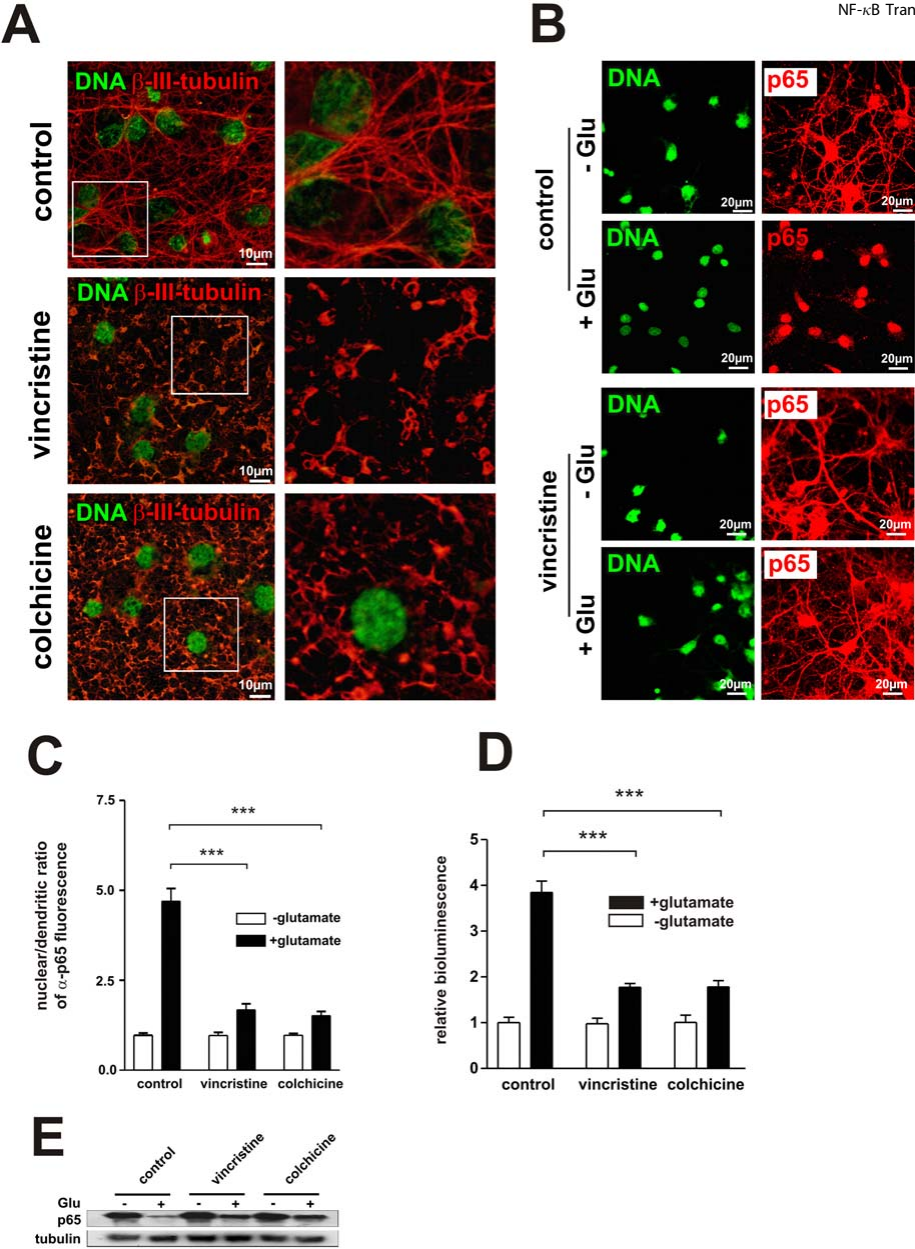
Microtubule-perturbing drugs inhibit neuronal transport of NF-κB. (A) Effect of microtubule-perturbing drugs on the organization of neuronal microtubule network. Microtubule network was visualized by α−tubulin immunostaining. Treatment of hippocampal neurons with 200 nM colchicine or 100 nM vincristine resulted in efficient depolymerisation, as shown by the diffuse tubulin staining and the loss of the well-organized microtubule patterns seen in untreated cells.(B) Hippocampal neurons treated with 300 µM glutamate for 5 min, either alone or after pre-treatment with 200 nM colchicine or 100 nM vincristine for 30 min were fixed (90 min after glutamate exposure) and visualised by SYTOX nuclear staining (green) and anti-NF-κB p65 immunofluorescence (red) to monitor neuronal transport of NF-κB (C) Quantification of nuclear/dendritic ratio of α-p65 fluorescence in neurons with functional or disrupted microtubules. Note that microtubule-perturbing drugs impaired dendritic to nuclear redistribution of NF-κB p65 after glutamate stimulation. (D) Reporter gene assay showed reduced NF-κB-dependent transcription activity in neurons with not functional microtubules. (E) In order to confirm the data obtained by microscopy, the subcellular localization of p65 was examined by cell fractionation and Western blotting. 250 µg of soluble protein from cytoplasmic extracts were immunoblotted for p65 protein level. In agreement, with the above experiments, pre-treatment with vincristine or colchicines before exposure to glutamate resulted in reduced relocation of p65 from cytoplasm to the nucleus.

Microtubules are components of the cytoskeleton and are serving as structural elements for the directed movement of the distinct motor protein families. Structurally, microtubules are linear polymers of α- and β-tubulin dimers. We used microtubule disrupting drugs vincristine and colchicine in order to suppress the dynein-dependent transport to study redistribution of NF-κB p65 from distal neuronal processes to the nucleus after glutamate stimulation. Fig 4A shows a microtubule network visualized by α−tubulin immunostaining. Treatment of hippocampal neurons with 200 nM colchicine or 100 nM vincristine resulted in efficient depolymerisation, as shown by the diffuse tubulin staining and the loss of the well-organized microtubule patterns seen in untreated cells. To determine whether dynein is involved in neuronal NF-κB transport, we stimulated neurons with glutamate (as described above) and then examined the subcellular localization of NF-κB p65 by immunostaining (Figure 4B). 90 min after glutamate stimulation, >80% of the cells displayed significant redistribution of p65 from neuronal processes and cell body to the nucleus. In contrast, pre-treatment of cultures with microtubule disrupting drugs resulted in the suppression of the p65 movement. We next quantified the average nuclear/dendritic ratio of p65 immunofluorescence intensity (Figure 4C). In order to confirm the data obtained by microscopy, the subcellular localization of p65 was examined by cell fractionation and Western blotting (Figure 4D). 250 µg of soluble protein from cytoplasmic extracts were immunoblotted for p65 protein level. In agreement with the above experiments, pre-treatment with vincristine or colchicine resulted in reduced relocation of p65 from cytoplasm to the nucleus after glutamate stimulation. To further characterise the effects of microtubule disruption on p65 transport, the NF-κB transcriptional activity was measured (Figure 4E). As expected, treatment with vincristine or colchicine reduced glutamate induced transcription activity. These results indicate that neuronal transport of NF-κB requires a functional microtubule network and implicate indirectly the involvement of the dynein.

## Material and Methods

### Plasmids

All plasmids were propagated using *E. coli* TOP10 (Invitrogen) and purified using Qiagen Mini-and Maxiprep kits (Qiagen). pNF-κB-Luc (Stratagene,) contains five repeats of a NF-κB-sensitive enhancer element upstream of the TATA box, controlling expression of Luciferase. To generate N-terminally his-tagged p65 construct a coding region of NF-κB p65 lacking translation stop codon was PCR-amplified from pBI-EGFP-TAP-p65 plasmid (a gift of Lienhard M. Schmitz, Giessen), cloned into pENTR/SD/D-TOPO vector using a pENTR Directional TOPO cloning kit, and subcloned into pET-DEST42 using Gateway System (Invitrogen). The NLS of the expression vector pET-DEST42-p65WT was mutated by site-directed mutagenesis PCR with following primers: forward primer 5′-GTCACCGGATTGAGGAGACACGTATAACGACATATGAGACCTTCAAGAGC-3′, reverse primer 5′-GCTCTTGAAGGTCTCATACGTCGTTGTACG TGTCTCCTCAATCCGGTGAC-3′. Clones were checked by restriction enzyme analysis and sequences were confirmed by automated DNA sequencing. C-terminal myc-tagged dynamitin expression vector (pCMVβ-Dynamitin) was a gift from Richard Vallee, Columbia University Medical Center.

### Immunoprecipitation from rat brain

Rat brains (Wistar strain) were homogenized in ice-cold solubilization buffer (50mM HEPES, 150 mM NaCl, 2 mM EGTA, 0.5% Triton X-100, 1 mM PMSF) in a Potter–Elvehjem homogenizer fitted with a motor-driven Teflon pestle and sonicated for 5 min to achieve better solubilization. Particulate matter was removed via centrifugation for 15 min at 35,000 g (4°C). Protein-protein interactions in the brain extract were stabilized prior to immunoprecipitation by treatment with the chemical cross-linking agent DSP (Pierce, Rockford, IL). For cross-linking, DSP was added to a final concentration of 0.5 mg/ml (freshly prepared in dimethylsulphoxide at 10 mg/ml). After 30 min on ice the reaction was stopped by the addition of Tris pH 8 (final concentration 25 mM). Brain extract (10 mg of total protein) was then incubated with 50 µl Protein G-Sepharose (Sigma; Deisenhofen, Germany) plus 30 µg monoclonal anti-NF-κB p65 antibody (sc-8008, Santa Cruz) or a non-immune mouse monoclonal IgG_1_ (clone MOPC 21, Sigma) as control for 2 hr with end-over-end rotation at 4°C. After five washes with 0.5 ml ice-cold solubilization buffer, the immunoprecipitated proteins were eluted from the beads via incubation with SDS-PAGE sample buffer and visualized via Western blot using appropriate antibodies.

### His-tag pull-down

*E. coli* BL21 (DE3) cells (Invitrogen) carrying the specified plasmid were grown in LB medium containing ampicillin (100 µg/ml) to 0.8 *A*_600 nm_, and the induction of recombinant protein expression was carried out by incubation with 1 mM Isopropyl-β-D-1-thiogalactopyranoside (IPTG) at 30°C for 4 h. Purification of recombinant proteins was performed using Ni-CAM affinity resin (Sigma; Deisenhofen, Germany) as recommended by the manufacturer. Briefly, the cells were harvested, resuspended in lysis buffer (10% glycerol, 0.3 M NaCl, and 50 mM Tris-HCl (pH 7.5)) incubated with lysozyme (1 mg/ml) for 30 min on ice, and briefly sonicated. The cell lysates were centrifuged, and the supernatant was applied to Ni-CAM equilibrated with binding buffer (500 mM NaCl, 20 mM Tris-HCl (pH 8.0), and 5 mM imidazole). The column was washed with washing buffer (500 mM NaCl, 20 mM Tris-HCl (pH 8.0), and 30 mM imidazole). The His-tagged protein was eluted with elution buffer (500 mM NaCl, 20 mM Tris-HCl (pH 8.0), and 1 M imidazole) and dialyzed against 20 mM Tris-HCl (pH 7.5) For the pull-down assays, the purified proteins (50 µg) were immobilized on 50 µl of Ni-CAM beads and incubated with 5 mg of pre-cleared brain extract prepared as described above in a total volume of 400 µl. After washing His-tagged proteins and their interactors were eluted from the beads by a 10 min incubation at room temperature in 20 µl of elution buffer as before. Pull-down eluates were analyzed by SDS-PAGE and immunoblotting with anti-IC74 (Chemicon, Hofheim, Germany) and anti-p150^Glued^ antibodies (BD Pharmingen, Heidelberg, Germany).

### Hippocampal neuron cultures and transfection

Primary cultures of rat hippocampal neurons were prepared from the hippocampi of E18-E19 Wistar rat embryos, after treatment with trypsin (0.2%, 15 min, 37°C; PAA) in Ca^2+^- and Mg^2+^-free Hank's balanced salt solution (HBSS; PAA). The hippocampi were then washed with HBSS containing 10% fetal bovine serum (PAA), to stop trypsin activity, and transferred to Neurobasal medium (GIBCO Invitrogen) supplemented with B27 supplement (1:50 dilution; GIBCO Invitrogen), 2 mM L-glutamine and 100 U/ml penicillin and 100 µg/ml streptomycin. The cells were dissociated in this solution and were then plated on ethylenimine-coated coverslips at a density of 45,000–50,000 cells/cm^2^. The cultures were maintained in a humidified incubator of 5% CO^2^/95% air, at 37°C, for eight days. Cultures were treated with 40 µM CNQX, 100 µM APV and 10 µM nimodipine for 24 h prior to start of experiments to establish a stable and low baseline of nuclearly localized NF-κB. Leptomycin B (Sigma; Deisenhofen, Germany) was dissolved in methanol and added to a final concentration of 20ng/ml (36 nM). For stimulations, neurons were washed and exposed to 300 µM glutamate (Sigma; Deisenhofen, Germany) for 5 min in the absence of these inhibitors at 37°C. After 5 min, the stimulus was washed out and cultures were returned to 37°C incubator for 90 min. When appropriate, 200 nM colchicine or 100 nM vincristine (Sigma; Deisenhofen, Germany), were added 30 min before stimulation, as indicated. Neurons were transfected using a Rat NSC Nucleofector Kit (Amaxa, Köln, Germany) according to the manufacturer's instructions with minor modifications. In particular, 5.0×10^6^ cells were used for each transfection. After dissociation of the neurons (see above), cells were centrifuged at 210 g for 10 min and re-suspended in an appropriate volume of Amaxa Nucleofector solution. After addition of DNA, the cells were electroporated in an Amaxa device and collected in 10 ml media. After further centrifugation at 210 g (10 min), the cells were re-suspended at appropriate density and then plated. Transfection efficiency was measured using the pmaxGFP vector (Amaxa) and analysis by fluorescence microscopy (Axiovert 100, Carl Zeiss, Jena).

### Confocal microscopy

Neurons were fixed in 3.7% PFA for 60 min at 4°C, permeabilized with 0.1% Triton X-100 in PBS for 5 min at room temperature, and then washed twice with PBS. To block non-specific antigenic sites, cells were incubated for 30 min with 5% goat serum (Jackson Immuno Research Laboratories, distributed by Dianova, Hamburg, Germany) in PBS. After washing in PBS, cells were incubated for 1h with primary antibodies in PBS, washed three times with PBS and finally incubated for 1h with secondary antibody and nuclear stain. The primary antibodies used were the mouse monoclonal anti-α-tubulin (DM1α; Sigma; Deisenhofen, Germany) diluted 1∶1000 and the rabbit polyclonal anti- NF-κB p65 antibody (sc-109; Santa Cruz Biotechnology, Inc., Santa Cruz, CA) diluted 1∶100. Detection was done with Cy3 conjugated antibody (1∶300, Jackson Immuno Research Laboratories, distributed by Dianova, Hamburg, Germany). Nuclear staining was done with SYTOX (1∶10000, Molecular Probes, Göttingen, Germany). Images were collected using a Zeiss Inverted Confocal Laser Scanning Microscope, LSM-Pascal (Zeiss, Jena, Germany); an excitation wavelength of 488 nm with an LP 505 emission filter, or wavelength of 543 nm with an LP 560 emission filter, was used to detect Cy3 or SYTOX, respectively. Images were analyzed using the LSM-510 Image software. Laser power and the detector settings were kept constant to maintain consistency in the data collection system. For localization studies, 15 or more cells pro condition were examined in random fields in at least three experiments. Non-neuronal cell contaminants in the culture were not counted. If the nucleus of a cell was condensed, fragmented, or perforated, the cell was considered as dead and was not analysed. Quantification of the fluorescence intensity was performed using Image J image analysis software (http://www.rsb.info.nih.gov/ij/). Statistical significance was determined by ANOVA with Bonferroni post hoc test, using GraphPad́s Prism. P<0.05 was considered significant.

### Preparation of cytoplasmic extracts

2×10^6^ neurons were washed twice in 10 ml cold PBS and resuspended in 200 µl of cytoplasmic extraction buffer (10mM HEPES, 1,5 mM MgCl_2_, 10 mM KCl, 0,5 mM DTT, 1mM PMSF, 1% NP-40; supplemented with Complete Mini Protease Inhibitor tablets) and incubated on ice for 5 min. The suspension was shortly vortexed and nuclei were pelleted at 1500g for 20 seconds. The supernatant containing cytoplasmic extract was collected and transferred to clean centrifuge tubes. Protein concentration was determined by Bradford assay (Bio-Rad Laboratories GmbH, Muenchen, Germany).

### Western blot analysis

A 250 µg portion of protein from immunoprecipitations, his-tag pull-downs or nuclear extracts were separated by SDS-PAGE on 12% polyacrylamide gels, electroblotted to PVDF membrane (Millipore Corporation Bedford, MA) and blocked in TBST+3% nonfat dry milk. The following first antibodies were used: mouse monoclonal anti-α-tubulin (B-5-1-2; Sigma; Deisenhofen, Germany), rabbit polyclonal anti-p65 NF-κB (sc 109; Santa Cruz Biotechnology). For detection horseradish peroxidase conjugated goat anti-rabbit and goat anti-mouse IgGs from Bio-Rad Laboratories GmbH (München, Germany) were used with an ECL kit (Amersham Pharmacia Biotech) according to manufacturer's protocol. To probe immunoblots with a second antiserum, membranes were stripped by incubation in 62.5 mM Tris-HCl, pH 6.7, 2% SDS, and 100 mM 2-mercaptoethanol for 30 min at 50°C. The blots were then incubated with antisera and processed as described above.

### Reporter gene assay

To detect NF-κB activity in glutamate-stimulated versus unstimulated hippocampal neurons, a Dual-Luciferase Reporter Assay System (Promega, Mannheim, Germany) and pNF-κB-Luc Reporter (Stratagene) was used. All cells were transfected with pNF-κB-Luc reporter vector and Renilla-luc control vector (Promega, Mannheim, Germany). When appropriate, dynamitin was cotransfected. As a mock vector, pMETalpha (Invitrogen) without insert was used. To quantify NF-κB-dependent transcription activity the cells were lysed and assayed for promoter-dependent luciferase activity versus promoter independent Renilla-luc activity (Lumat LB9507 device, Berthold Technologies, Bad Wildbach, Germany).

## Discussion

**Figure 5:**
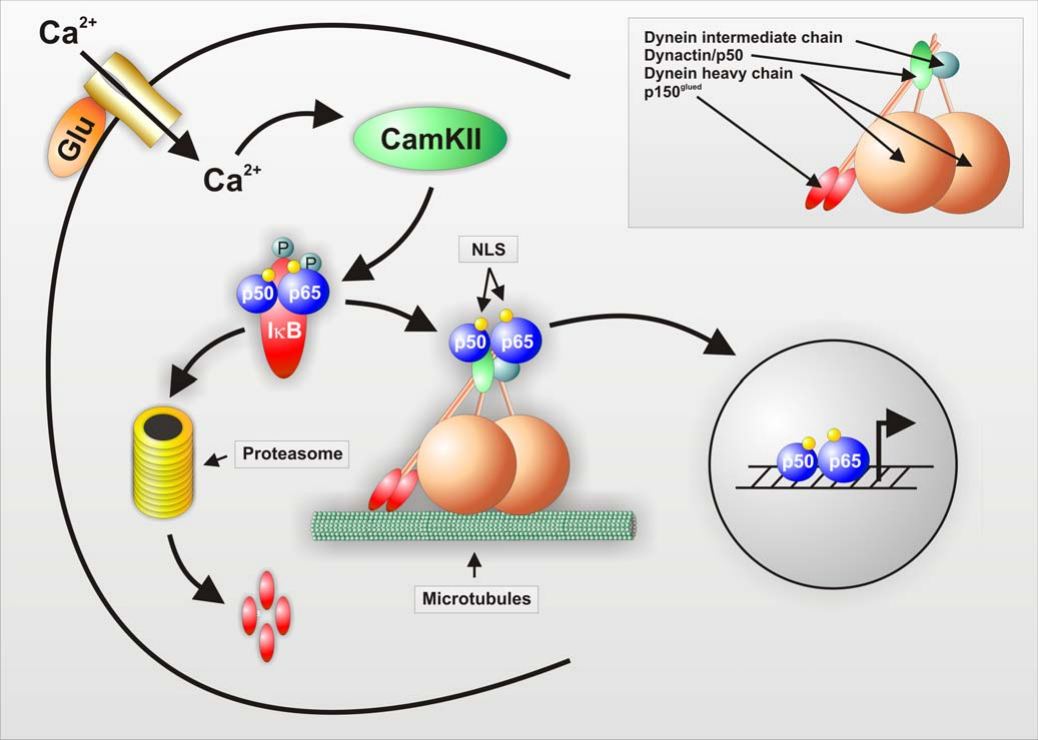
Schematic presentation for the NF-κB activation by synaptic activity and its dynein-mediated retrograde transport to the nucleus along microtubules. Upon stimulation of primary neurons with glutamate, different signalling pathways (represented here by CaMKII) originating from intracellular Ca^2+^ elevation induces phosphorylation of IκB, which subsequently leads to its degradation within the proteasome. Thereby, the nuclear localisation signals (NLS) of NF-κB subunits are unmasked, allowing its binding to importin α/β heterodimer. This complex is then transported retrogradely towards the nucleus via an association with motor protein dynein/dynactin, where it activates NF-κB target genes.

In this study, we demonstrate cytoplasmic dynein to be the motor protein transporting NF-κB from distant neuronal processes, including synaptic compartments, to the nucleus (Figure 5). Cytoplasmic dynein is a minus end-directed, microtubule-based motor. Each cytoplasmic dynein molecule is composed of two catalytic heavy chains (HCs 1, 532 kD) exhibiting both ATPase and motor activities and accessory subunits including several intermediate chains (ICs, 74 kD), light intermediate chains (LICs, 53-59 kD) and light chains (LCs, 10-14 kD). (King, 2000) Cytoplasmic dynein requires another multisubunit protein complex, dynactin, for full activity [41]; [42]. It is composed of: p150^Glued^, which exhibits extensive sequence homology with the product of the Glued gene in Drosophila [43]; Arpl, a novel actin-related protein; dynamitin; and several uncharacterized subunits. Dynactin has been proposed to activate the motor function of dynein by increasing the processivity of the motor [44]. It also participates in cargo binding [45]. We used co-immunoprecipitation and cross-linking experiments to verify the interaction of NF-κB and cytoplasmic dynein *in vivo*. The cross-linking reagent DSP was successfully used in a variety of applications for identification of protein-protein interactions [46]–[49]. We found dynein intermediate chain (IC74) and two subunits of dynactin dynamitin and p150^Glued^ to be present in complex with NF-κB. Additionally, using *in vitro* protein pull-down experiments, we showed that this complex formation requires functional NLS, which is present in NF-κB p65.

There exist several prior indications of microtubule involvement in dynein-mediated transport of NLS-containing protein cargos. In *Aplysia* neurons it has been shown that microtubules are required for retrograde transport of NLS-labeled proteins along the axon [50]. In mammalian neurons microtubules were shown to be involved in retrograde transport of nuclear import complexes within the axon after injury [37]. Salman et al. have shown that NLS-containing peptides induced molecular delivery along microtubules in an extract of *Xenopus laevis* eggs [51]. The nuclear transport of the parathyroid hormone (PTH)-related protein was reduced in cells with microtubules depolymerized by nocodazole [52]. The tumour suppressor protein p53 was found to be associated with microtubules and to require dynein for its nuclear import [53]. Gene delivery vectors containing repetitive binding sites for NF-κB were transported along microtubules in a dynein-dependent manner [54]. Given the above, we used microtubule-perturbing drugs to inhibit neuronal transport of NF-κB. Hippocampal neurons exposed to vincristine or colchicine showed reduced mobility of activated NF-κB towards the nucleus and lower NF-κB-dependent transcription activity after glutamate stimulus. In contrast, non-neuronal tumor cell lines do not require the cytoskeleton to transport activated NF-κB towards the nucleus [55].

Dynein and dynactin function can be inhibited by blocking their interaction. Overexpression of the dynactin subunit dynamitin destroys dynactin function by releasing its dynein-binding p150^Glued^ subunit [56], [57]. These effects are proposed to be the result of competitive inhibition of the dynein-dynactin interaction by excess free dynein-binding polypeptide [58]. Mice with a mutation in the dynein heavy chain (*Dnchc1*) show defects in retrograde axonal transport and motoneuron survival [59]. Inhibition of dynein by postnatal overexpression of the motor-protein dynamitin also results in a significant inhibition of microtubule based retrograde transport [60]. To test whether cytoplasmic dynein is the motor that transports NF-κB we inhibited cytoplasmic dynein function by disrupting dynactin. Our data show that overexpression of dynamitin in hippocampal neurons prevents nuclear accumulation of p65 and reduces NF-κB-dependent activity. Taken together this study shows to our knowledge for the first time molecular mechanism for the retrograde transport of activated NF-κB from distant synaptic sites towards the nucleus.
